# Retrospective review of metastatic hormone receptor-positive inflammatory breast cancer patients reveals poor responses to cyclin dependent kinase 4/6 inhibition

**DOI:** 10.1186/s13058-025-02162-y

**Published:** 2025-12-10

**Authors:** Azadeh Nasrazadani, Rebecca S. Tidwell, Megumi Kai, Bora Lim, Vicente Valero, Debu Tripathy, Sadia Saleem, Bisrat G. Debeb, Anthony Lucci, Wendy A. Woodward, Rachel M. Layman, MDACC IBC Clinic and Research Team, Rachel Layman, Michael C. Stauder, Susie X. Sun, Gary J. Whitman, Miral Patel, Huong Le-Petross, Yang Lu, Angela Marx, Angela Alexander, Chasity Yajima, Lily Villareal, Heather Lopez

**Affiliations:** 1 Department of Breast Medical Oncology, The University of Texas MD Anderson Cancer Center, 1515 Holcombe Blvd, Houston, TX 77030 USA; 2 Morgan Welch IBC Clinic and Research Program, The University of Texas MD Anderson Cancer Center, Houston, TX USA; 3 Department of Biostatistics, The University of Texas MD Anderson Cancer Center, Houston, TX USA; 4 Department of Surgical Oncology, The University of Texas MD Anderson Cancer Center, Houston, TX USA; 5 Department of Breast Radiation Oncology, The University of Texas MD Anderson Cancer Center, 1515 Holcombe Blvd, Houston, TX 77030 USA

**Keywords:** CDK, Palbociclib, Ribociclib, Abemaciclib, Inflammatory breast cancer, First-line, Metastatic

## Abstract

**Background:**

Patients with inflammatory breast cancer (IBC) have aggressive biology and relatively inferior responses to standard-of-care (SOC) therapies. Understanding the efficacy of SOC therapies in IBC is critical to optimize outcomes. Our objective was to assess the progression-free survival (PFS) of metastatic hormone receptor-positive HER2-negative/low (HR+HER2−) IBC patients treated with CDK4/6 inhibitors (CDKIs) and hormonal therapy (HT).

**Methods:**

Data from 58 IBC patients with metastatic HR + /HER2- IBC from a single institution were reviewed. The medians (95% confidence intervals) of overall survival (OS), PFS, and time on treatment (ToT) from the time of CDKI initiation were reported via the Kaplan‒Meier method. Differences were tested by the log-rank test.

**Results:**

We identified 58 patients (including 16 with de novo stage IV disease). The median OS, PFS, and ToT in the total cohort were 26 (16, 37), 7 (5, 10), and 7 (5, 10) months (mos), respectively. No differences were observed between pre-menopausal patients and postmenopausal patients. The OS, PFS, and ToT rates at the initial diagnosis of Stage III later developing metastatic breast cancer (MBC, N = 42) and de novo IV (N = 16) patients were 19 (15, 34) vs 34 (21, NR), 7 (5, 14) vs 9 (6, NR), and 6 (5, 10) vs 9 (4, NR) mos, respectively (ns). OS, PFS, and ToT in patients receiving CDKI in the first-line vs second-line metastatic setting were 27 (19, 44) vs 17 (12, 39), 7 (5, 15) vs 6 (3, NR), and 7 (5, 15) vs 6 (3, 20) mos, respectively (ns). Among the patients initially diagnosed with stage III disease later progressing to MBC, brain metastases were observed in 12/42 patients. Thirty-eight patients underwent genomic testing either before CDKI treatment (N = 21) or at progression (N = 17). Among the 38 patients who underwent genomic testing, 34 had mutations, most commonly in TP53, PIK3CA, FGFR1, CCND1, and ARID1A. ESR1 mutations were present in 0% of the samples tested prior to CDKI treatment, and 29% of the samples tested at progression.

**Conclusions:**

Patients with metastatic HR+HER2− IBC demonstrated a shorter time on treatment suggesting shorter duration of response on CDKI + HT, which is markedly inferior to reported data for non-IBC patients from phase III trials.

## Background

Inflammatory breast cancer (IBC) is an uncommon subtype of breast cancer diagnosed based on presenting symptoms of rapidly developing, diffuse breast skin edema and discoloration and pathologic confirmation of breast cancer [1, 2]. Owing to challenges in diagnosis [3] and the rapid progression of disease, approximately one-third of patients are diagnosed at stage IV [4]. Patients with IBC face a dismal prognosis because of the aggressive nature of the disease and suboptimal response to standard therapies [4]. At presentation, hormone receptor-negative HER2-negative/low (HR-HER2-) and HER2-positive (HR-HER2 +) breast cancer subtypes occur with greater frequency among IBC patients than among non-IBC patients. However, the hormone receptor-positive HER2-negative/low (HR+HER2−) subtype is still the most common subtype in IBC patients, as it is common in non-IBC patients; however, unlike non-IBC patients, HR + IBC is not associated with an indolent course or favorable outcomes [5–8]. Similarly, among metastatic IBC patients, the HR+HER2− subtype is not associated with an indolent course and indeed has similar or worse overall survival (OS) compared to triple positive or HR-HER2 + patients [9].

Cyclin-dependent kinase 4/6 inhibitors (CDKIs) have shown promise in the treatment of HR+HER2− metastatic breast cancer (MBC) [10–17], but their efficacy in treating inflammatory MBC remains unclear. Owing to the morbidity of local progression in IBC, de novo metastatic IBC is often treated with neoadjuvant systemic therapy, surgery and radiation such that CDKIs are offered in the pseudoadjuvant setting to delay progression or prevent recurrence when no evidence of disease (NED) is achieved [18, 19] in the first-line or second-line metastatic setting. Here, we present the outcomes of patients with IBC treated with CDKIs in the metastatic setting.

Our results indicate that patients with metastatic IBC exhibit a poor response to CDK inhibition, with short median progression-free survival (PFS) and OS compared with previous reports in non-IBC patients [11, 13, 15, 20]. These findings suggest that the aggressive biology of IBC may limit the efficacy of this targeted therapy approach.

## Methods

### Study population

This was a single-center retrospective study utilizing a prospectively maintained IBC registry at The University of Texas MD Anderson Cancer Center (MDACC). Patients enrolled to the IBC registry are enrolled to either cohort 1, whom are diagnosed with IBC based on consensus evaluation in the multidisciplinary IBC clinic and are untreated, or to cohort 2, whom have previously been diagnosed by their local referring physician prior to evaluation at MDACC and have started treatment. Patients with HR+HER2− IBC for which CDKI was administered in the metastatic setting (cohort 1 and 2) were included in this study (N = 56). Clinicopathologic, reproductive, treatment, and outcome data were extracted from the IBC registry database (2006–1072). Clinically ordered genetic testing results were extracted from the charts. Chart review was performed by MK in a systematic manner to ensure consistent and accurate data collection. In cases of incomplete data, additional retrospective chart reviews were conducted. This study was approved by the Institutional Review Board at The University of Texas MD Anderson Cancer Center (Protocol PA17-0274).

### Endpoints

The time on treatment (ToT), PFS, and OS are reported from the time that CDKI treatment is started. ToT represents number of months patients remained on therapy versus PFS which specifies time until progression of disease or death.

### Sample size and statistical analyses

Descriptive statistics were used to analyze patient characteristics. Clinical and biological variables were grouped into standard categories whenever reasonable. Continuous variables are expressed as medians and interquartile ranges (IQRs). Categorical variables are expressed as numbers and proportions (%) and were compared via Fisher's exact test.

PFS and OS were calculated via the Kaplan‒Meier method and compared via log-rank tests. The median follow-up was calculated by reversing the censor for the Kaplan‒Meier estimate for OS. All tests were two-sided at a significance level of α = 0.05. Statistical analyses were performed with SAS software, version 18 (StataCorp, TX, USA).

## Results

Forty-two patients were stage III at presentation and relapsed after multidisciplinary therapy. Sixteen patients presented with de novo stage IV disease. From the original N = 58 cohort, 2 patients were censored in the CDKI-stratified analysis due to a history of use of > 1 CDKI due to toxicity or intolerance. Baseline demographics and clinicopathologic characteristics are reported in Table 1. The median age at diagnosis was 52 years. The majority of the cohort was non-Hispanic and white. Sixty-four percent were postmenopausal, and 67% had first-line CDKI.

**Table 1:** Patient and disease baseline characteristics

Age at Dx, Median (IQR)	52.5 (41.0, 62.0)
Race, n (%)	
White	50 (86.2%)
Black or African American	2 (3.4%)
Native Hawaiian or other Pacific Islander	1 (1.7%)
Asian	3 (5.2%)
Other	2 (3.4%)
Ethnicity, n (%)	
Hispanic or Latino	4 (6.9%)
Not Hispanic or Latino	53 (91.4%)
Unknown	1 (1.7%)
Year of Dx, n (%)	
2007	2 (3.4%)
2009	2 (3.4%)
2011	1 (1.7%)
2012	2 (3.4%)
2013	4 (6.9%)
2014	3 (5.2%)
2015	2 (3.4%)
2016	9 (15.5%)
2017	7 (12.1%)
2018	2 (3.4%)
2019	6 (10.3%)
2020	6 (10.3%)
2021	8 (13.8%)
2022	3 (5.2%)
2023	1 (1.7%)
Menopausal staus, n (%)	
Postmenopause	37 (63.8%)
Premenopause	21 (36.2%)
Initial clinical stage, n (%)	
III	42 (72.4%)
IV	16 (27.6%)
Histology, n (%)	
IDC	45 (77.6%)
ILC	5 (8.6%)
mDLC	7 (12.1%)
Invasive, not specified	1 (1.7%)
CDKI agent, n (%)	
Palbociclib	36 (62.1%)
Ribociclib	9 (15.5%)
Abemaciclib	11 (19.0%)
Other	2 (3.4%)
Line of therapy utilized, n (%)	
1st	39 (67.2%)
2nd	19 (32.8%)

**Fig. 1:**
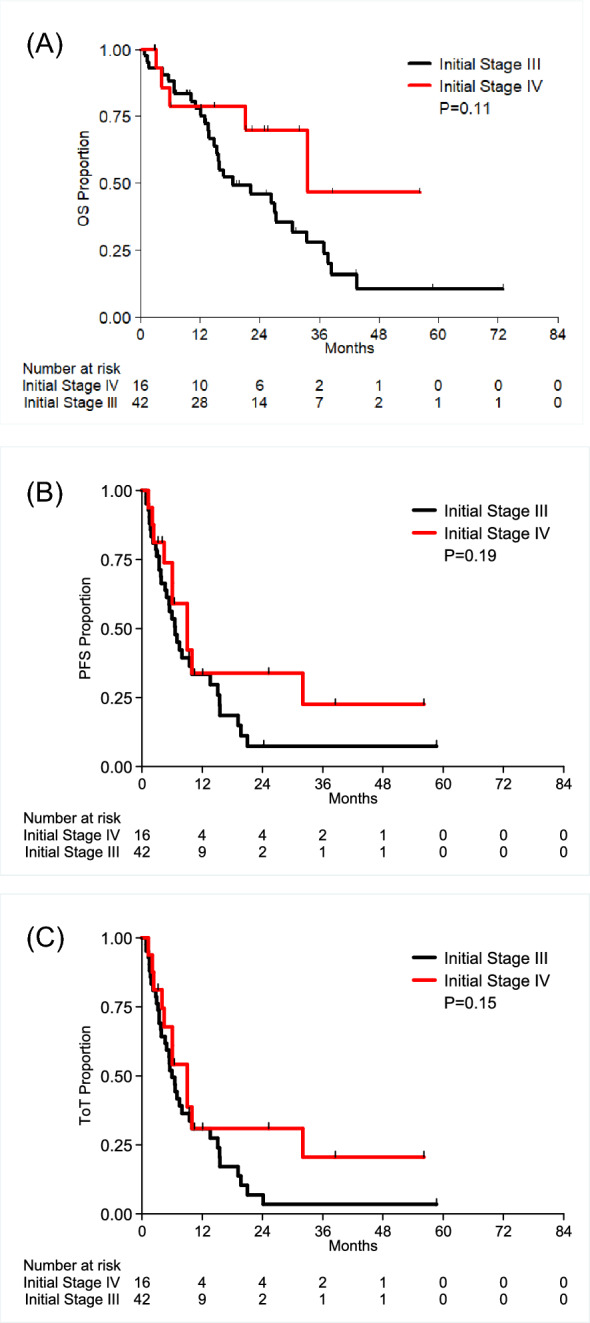
K‒M curves for cohort outcomes. **A** OS of patients with metastatic HR + HER- IBC utilizing the CDKI stratified by stage at the time of diagnosis (ns, top). **B** Progression-free survival of patients with metastatic HR + HER- IBC utilizing the CDKI stratified by stage at the time of diagnosis (ns, middle). **C** Time to treatment of patients with metastatic HR + HER- IBC utilizing the CDKI stratified by stage at the time of diagnosis (ns, bottom)

After a median follow-up time of 17 months, the median OS, PFS, and ToT in the total cohort were 26 (16, 37), 7 (5, 10), and 7 (5, 10) months (mos), respectively (Table 2). No differences were observed between pre- and postmenopausal patients. The OS, PFS, and ToT rates of patients with initial Stage III (N = 42) vs IV (N = 16) disease were 19 (15, 34) vs 34 (21, NR), 7 (5, 14) vs 9 (6, NR), and 6 (5, 10) vs 9 (4, NR) mos, respectively (ns) (Table 2, Fig. 1a–c).

**Table 2:** Outcome by treatment

	CDKI			Total (N = 56)	*P* value
	Abemaciclib (N = 11)	Palbociclib (N = 36)	Ribociclib (N = 9)		
OS (months)					0.67
Events/N	6/11	26/36	2/9	34/56	
Median (95% CI)	16.72 (10.28–NE)	26.35 (15.74–37.03)	NE (11.27–NE)	26.35 (15.84–37.03)	
12 months Est (95% CI)	71.59% (48.84–100.00)	80.56% (68.61–94.58)	70.00% (42.01–100.00)	77.73% (67.34–89.73)	
24 months Est (95% CI)	47.73% (24.11–94.48)	50.54% (36.22–70.53)	70.00% (42.01–100.00)	50.84% (38.31–67.47)	
PFS (months)					0.23
Events/N	6/11	32/36	5/9	43/56	
Median (95% CI)	6.97 (3.42–NE)	5.98 (4.40–9.95)	9.03 (7.49–NE)	6.64 (5.42–9.99)	
12 months Est (95% CI)	47.73% (24.11–94.48)	24.55% (13.54–44.51)	46.67% (21.05–100.00)	31.48% (20.76–47.75)	
24 months Est (95% CI)	23.86% (5.09–100.00)	9.21% (3.14–26.96)	23.33% (4.72–100.00)	13.12% (5.96–28.86)	
ToT (months)					0.29
Events/N	8/11	34/36	5/9	47/56	
Median (95% CI)	6.05 (3.42–NE)	5.55 (4.40–9.46)	9.03 (7.49–NE)	6.05 (4.93–9.46)	
12 months Est (95% CI)	36.36% (16.64–79.47)	23.19% (12.66–42.48)	46.67% (21.05–100.00)	28.89% (18.83–44.31)	
24 months Est (95% CI)	18.18% (3.70–89.26)	8.70% (2.95–25.61)	23.33% (4.72–100.00)	12.04% (5.44–26.64)	
pCR, n (%)					0.12
pCR	1 (11.1%)	0 (0.0%)	1 (20.0%)	2 (5.1%)	
nonpCR	8 (88.9%)	25 (100.0%)	4 (80.0%)	37 (94.9%)	
Missing	2	11	4	17	
Developed metastases (III), n					
Yes	9	28	5	42	
Brain mets (III), n (%)					0.06
No brain mets on file	7 (77.8%)	19 (67.9%)	4 (80.0%)	30 (71.4%)	
1st site	2 (22.2%)	0 (0.0%)	0 (0.0%)	2 (4.8%)	
Later time	0 (0.0%)	9 (32.1%)	1 (20.0%)	10 (23.8%)	
Brain mets as first site (III), n (%)					0.02
1st site	2 (100.0%)	0 (0.0%)	0 (0.0%)	2 (4.8%)	
Later time	0 (0.0%)	9 (100.0%)	1 (100.0%)	10 (23.8%)	

NR = Not reached

Patients treated with abemaciclib (N = 11), palbociclib (N = 36), and ribociclib (N = 9) experienced PFS rates of 7 (3, NR), 6 (4, 10), and 9 (7, NR) mos, respectively, and a ToT of 6 (3, NR), 6 (4, 9), and 9 (7, NR) mos, respectively (Table 2). Two patients were excluded from the original cohort in this analysis because either the CDKI utilized was not known or if more than one CDKI was utilized. OS, PFS, and ToT in patients receiving CDKI in the first-line vs second-line metastatic setting were 27 (19, 44) vs 17 (12, 39), 7 (5, 15) vs 6 (3, NR), and 7 (5, 15) vs 6 (3, 20) mos, respectively (ns). Among patients initially diagnosed at stage III, brain metastases were observed in 12/42 patients. Notably, of the 12 patients who developed brain metastasis, 9 patients were treated with palbociclib (Table 2).

Genomic testing data were available for 38 patients and examined by timing pre-CDKI (N = 21) or post-CDKI (N = 17). Tumor mutations were identified in 34/38 patients (89%). The most frequently mutated genes, including TP53, PIK3CA, FGFR1, CCND1, and ARID1A, were present regardless of the timing of testing (Table 3). Alterations span key oncogenic pathways, such as the PI3K/AKT/mTOR pathway, RTK signaling, DNA damage repair, and chromatin remodeling. Mutations that occurred only pre-CDKI or post-CDKI testing were more sporadic (Table 4), but five ESR1 mutations were present only post-CDKI (5/17 = 29% post-CDKI results).

**Table 3:**
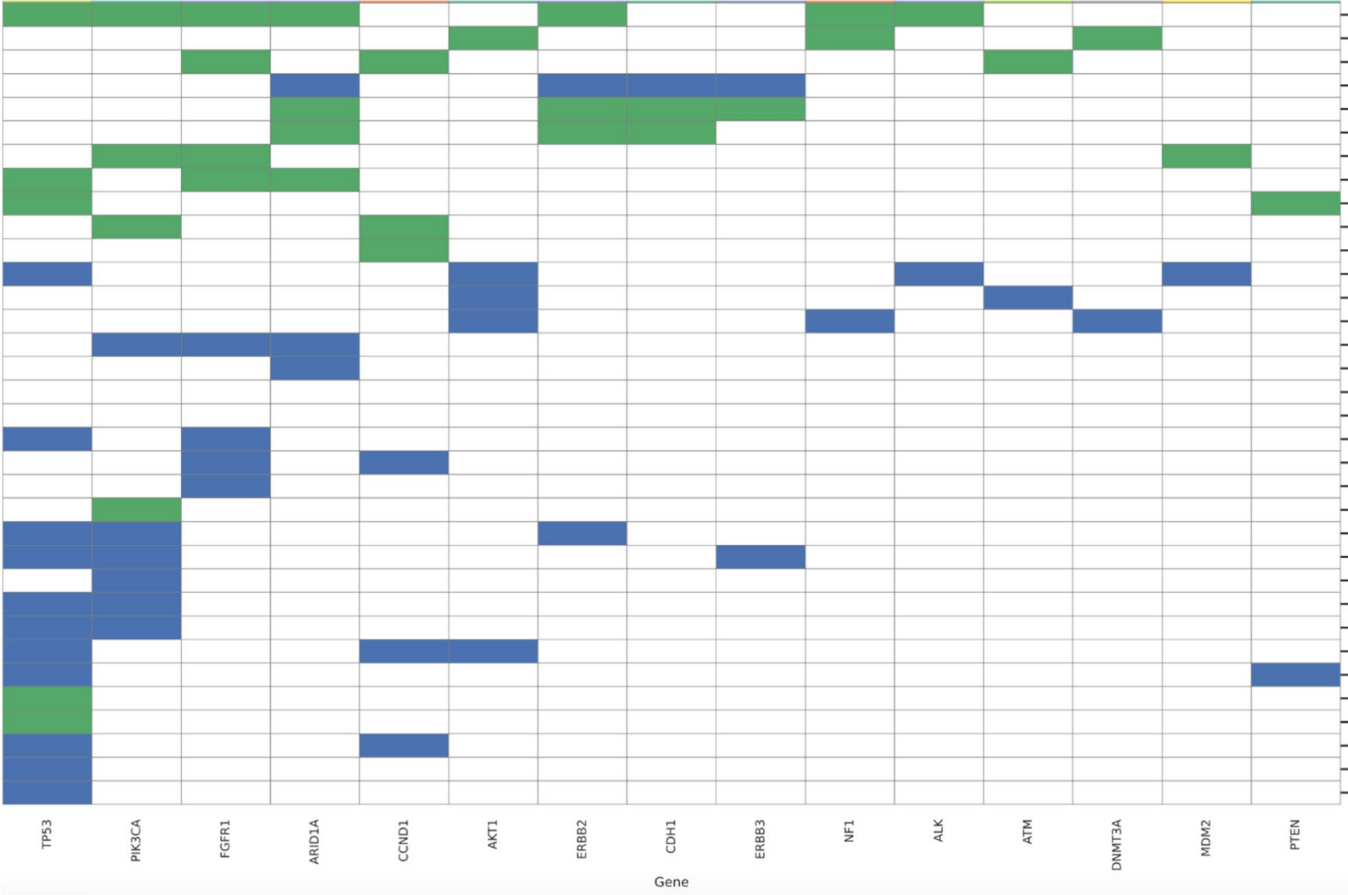
Genomic mutations identified in both pre-CDKI (blue) and post-CDKI (green) results. Each row represents one case.

**Table 4:**
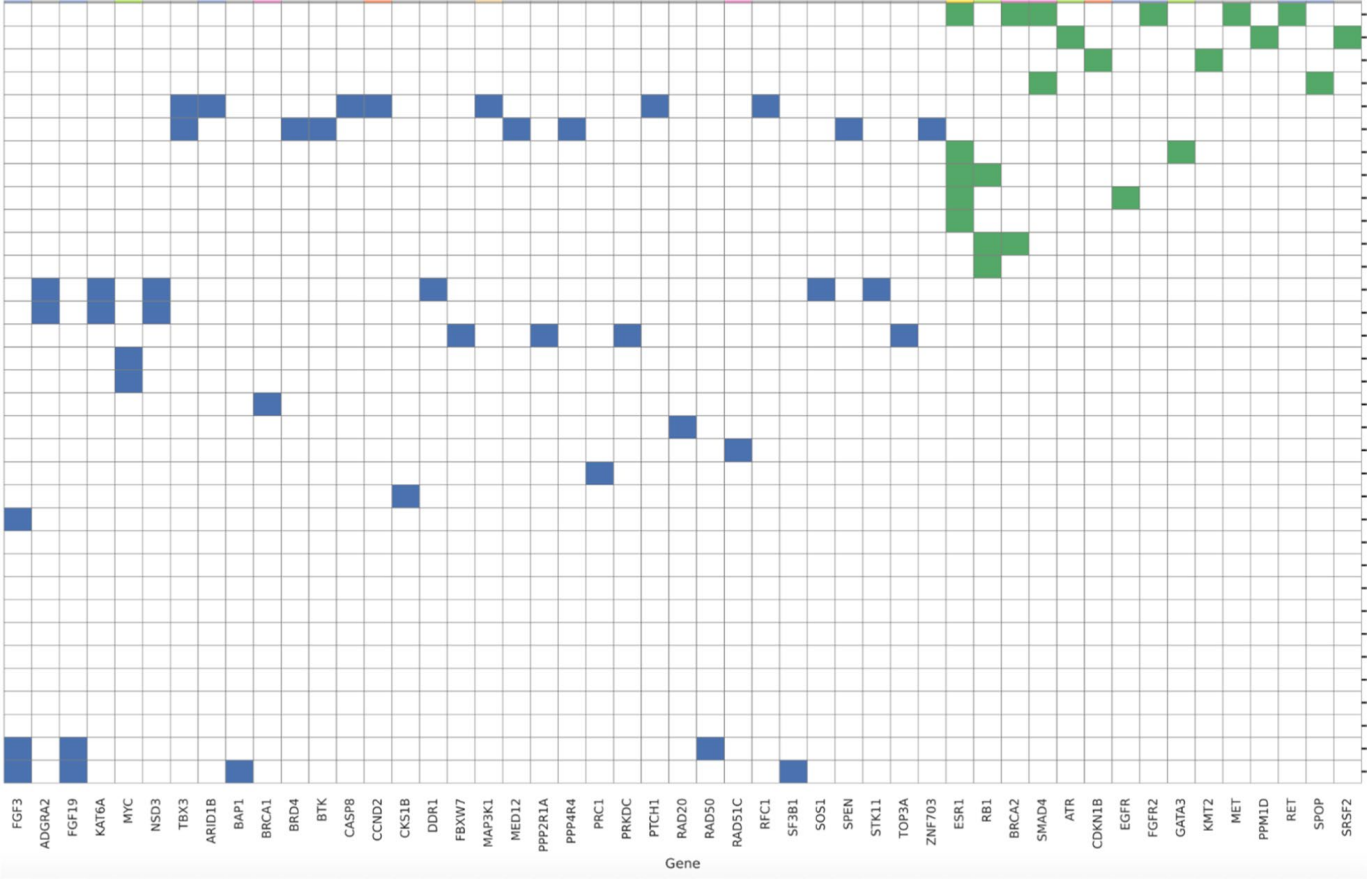
Genomic mutations identified only in either pre-CDKI (blue) or post-CDKI (green) testing. Each row represents one case.

## Discussion

IBC patients were excluded from randomized trials demonstrating the efficacy of abemaciclib and ribociclib CDKIs in HR + HER- MBC [11, 13–15]. While patients with IBC were allowed to enroll in PALOMA 3 [20], which utilizes palbociclib in this space, data focused on this population are not reported. Thus, there are no data on the efficacy of CDKIs in metastatic IBC patients. Our study revealed that patients with metastatic HR+HER2− IBC demonstrate a poor response to CDKI-based therapy and have a disproportionately high risk of brain metastasis. Patients receiving CDKI in the first- versus second-line setting demonstrated relatively superior outcomes. Overall, however, outcomes are significantly inferior to historical data compared with non-IBC data [10–16].

Among women with HR+/HER2− MBC, multiple randomized clinical trials have demonstrated a significant improvement in PFS following the addition of CDKI to standard HT. These trials studied palbociclib (PALOMA-2 and -3) [10, 20], abemaciclib (MONARCH-2 and -3) [14, 15], and ribociclib (MONALEESA-2, -3 and -7) [12, 13]. Notably, ribociclib has been shown to improve overall survival in these trials [11, 12], indicating critical advancements in treatment. The median progression-free survival (PFS) exceeded or approached two years in MONARCH-3, MONALEESA-2, 3 and -7, providing a benchmark for the expected efficacy of these therapies, against which PFS in patients with metastatic IBC falls short by more than 50% on the basis of our findings. Several studies have attempted to define the molecular landscape of IBC and have identified preferentially activated pathways, including CTNB, HER2, MYC, RAS, IFN-a, IFN-g, TNF-a, and VEGF [21]. A robust, distinctive IBC signature, however, has not been identified [22]. Despite the lack of a clear unique signature for IBC, a greater propensity for gene clustering, which is consistent with more aggressive basal-like or ErbB2 molecular subtypes, has been reported, with less frequent IBC tumors demonstrating luminal A subtype gene clustering [23]. The proportion of luminal A IBCs was reported to be as low as 7.7% in one study [24], which is significantly lower than the approximately 30% [8] expected to demonstrate HR+HER2− histopathology, suggesting a molecularly more aggressive phenotype in HR+HER2− IBCs than would otherwise be expected in non-IBC breast cancers. Congruently, pooled analysis of the MONALEESA 2, 3, and 7 trials demonstrated an increased risk of death for patients receiving ribociclib + HT harboring luminal B, ErbB2 and basal-like subtypes compared with luminal A subtype tumors [25], highlighting the variability in sensitivity and responsiveness to CDKI-based approaches in HR+HER2− MBC, which inevitably leads to resistance to therapy.

Furthermore, while the potential brain penetration of at least abemaciclib has been studied (reviewed in [26]), the incidence of brain metastases in this population remains higher than expected. Interestingly, among the patients who developed brain metastases, the majority had received palbociclib, which should be considered with caution given that this was the first approved CDKI and thus represents a larger sample size and longer follow-up.

Genomic testing revealed mutations in the majority of patients. This is the first report to examine mutations specifically in HR + IBC patients exposed to CDKI and demonstrates rates of acquired ESR1 mutations comparble to those reported in non-IBC [27, 28] and multiple actionable mutations, including BRCA. Similarly, we report RB1 somatic abberations in 3/34 (~ 8.8%) cases exclusively in the post treatment setting in line with that reported in the literature in CDKI resistance breast cancer [29, 30]. Given the highly dependent nature of CDKIs on intact RB1, treatment resistance is expected with mutations leading to RB1 loss or impaired binding to partners. Interestingly, preclinical CDKI resistant MCF-7 and KPL-1 cell lines were found to retain sensitivity to cytotoxic chemotherapy agents and without affecting ERα expression levels [31], reinforcing inevitable and earlier transition to chemotherapy rather than shift to alternative endocrine therapy combinations with more favorable side effect profiles. Notably enriched within the next generation sequencing data are highly frequent aberrations noted in TP53 (16/34), and collectively in PIK3CA(10/34), AKT(5/34), and PTEN(2/34) genes both in pre and post treatment tumors. TP53 and PIK3CA pathway mutations driving tumor resistance are well reported in the literature and suspected to be contributing to a higher degree in IBC as compared to lesser incident ESR1 driver mutations. Targeted therapies recently approved and in trial may elucidate individual contribution of these pathways and whether resistance may be overcome depending on timing and sequencing of such novel monotherapies or combinations.This work has several limitations. Perhaps most importantly, the incorporation of these agents in the treatment of IBC is relatively recent, and as such, sample size and follow-up time are limited despite representing the only and largest cohort of its kind to date. For this reason, comparisons between the agents are not feasible. Sequential approval of individual CDKIs and thus variation in the time frame in which CDKIs are received for these patients may be associated with confounding factors, further limiting analysis between agents. Importantly, the follow-up time is adequate given the limited efficacy of the drugs and the unfortunate low duration of benefit derived. Finally, limitations inherent to retrospective chart review are anticipated; however, at this time, this is the highest level of data available. Despite the suboptimal outcomes reported herein with CDKIs in HR+HER2− IBC, the combination of CDKI and HT improves upon HT monotherapy and provides significant clinical benefit in a subset of IBC patients (data not shown) for which omission of these agents cannot be justified. While CDKI and HT remains the SOC for patients with metastatic HR+HER2− breast cancer, inclusion of patients with IBC in key clinical trials is crucial to ensure findings are generalizable to patients with this uniquely aggressive biology. Enhanced understanding of underlying resistance mechanisms are essential to achieiving advances in outcomes in IBC.

## Conclusion

We found significantly inferior oncological outcomes, including median PFS and OS, with CDKI-based therapy in the metastatic setting for the management of IBC than with those reported in large-scale studies in patients with non-IBC HR+HER2− MBC, suggesting the approval of these agents. Our work highlights the urgent need for more efficacious therapies for patients with IBC. Translational work to understand the mechanisms of resistance is integral to guiding these efforts and identifying more effective treatment strategies for this challenging disease.

## Data Availability

Deidentified data used in this study are available from the corresponding author upon reasonable request.

## Abbreviations

IBC: Inflammatory breast cancer
SOC: Standard of care
PFS: Progression-free survival
HR+HER2−: Hormone receptor-positive HER2 negative/low
CDKIs: CDK 4/6 inhibitors
HT: Hormonal therapy
OS: Overall survival
ToT: Time on treatment
MBC: Metastatic breast cancer
MDACC: The University of Texas MD Anderson Cancer Center
